# Oncological Outcome of Primary and Secondary Muscle-Invasive Bladder Cancer: A Systematic Review and Meta-analysis

**DOI:** 10.1038/s41598-018-26002-6

**Published:** 2018-05-15

**Authors:** Peng Ge, Li Wang, Meng Lu, Lijun Mao, Wang Li, Rumin Wen, Jian Lin, Junqi Wang, Jiacun Chen

**Affiliations:** 1 Department of Urology, the Affiliated Hospital of Xuzhou Medical University, Xuzhou, Jiangsu China; 2 Xuzhou Medical University, Xuzhou, Jiangsu China; 3 Department of Urology, Peking University First Hospital and Institute of Urology, Peking University, Beijing, China; 4 National Research Center for Genitourinary Oncology, Beijing, China

## Abstract

Conflicting results of survival outcomes for primary and secondary muscle-invasive bladder cancer (MIBC) have been reported in previous studies. Primary MIBC is defined as presentation of muscle-invasive disease at initial diagnosis while secondary MIBC presumes that non-muscle invasive disease later progressed to MIBC. Due to the varying reports, we conducted a systematic review and meta-analysis to compare survival outcomes between the two groups. Relevant studies were retrieved from Medline, Embase, the Cochrane Library, and Scopus using a comprehensive search approach. Cancer-specific survival (CSS) was the outcome measure. A total of 14 studies involving 4,075 cases were included. Patients with secondary MIBC were significantly correlated with worse CSS in model I (pooled HR: 1.29, 95% CI: 1.07–1.56, *P* = 0.008). The results of sensitivity analyses indicated that the omission of any single study each time did not have a significant impact on the combined risk estimates. Egger’s test suggested no publication bias among these studies. The European Organization for Research and Treatment of Cancer (EORTC) risk score offers the possibility of stratifying the secondary MIBC patients into different risk groups. In high-risk NMIBC, timely radical cystectomy should be considered. Further study is required to assess the multimodal therapy in both high-risk NMIBC and secondary MIBC patients as well as to evaluate genetic and molecular drivers of tumor induction, promotion, and progression.

## Introduction

There will be an estimated 79,030 new cases and 16,870 deaths from bladder cancer in the United States in 2017^1^. Roughly three quarters of cases are diagnosed as non-muscle invasive bladder cancer (NMIBC)^2,3^. Tumor recurrence in NMIBC is quite common and, among the high-risk subgroup, up to 50% will progress to muscle invasive bladder cancer (MIBC)^4^.

For patients with non-high risk NMIBC, conservative therapy with closer surveillance is the preferred option. However, when the disease develops to invade the detrusor muscle, radical cystectomy (RC) with pelvic lymphadenectomy is the gold standard. Among patients treated with RC because of MIBC, 57% have muscular invasion at first presentation (primary MIBC), and the remainder have a history of NMIBC that subsequently progressed to MIBC despite organ-preserving treatment (secondary MIBC)^5^. In normal daily practice, patients with primary and secondary MIBC are both offered RC equally. However, the oncological outcome of these patients remains in debate with conflicting results in the literature^3,4,6–21^.

Secondary MIBC compared to primary MIBC could convey superior outcome because of the initial non-muscle invasive bladder cancer character or likewise convey inferior outcome because of its progressive tumor biology^7,8,13,22^. Meanwhile, similar prognosis was also reported in some studies^10,11^.

Currently, indications for RC for patients with NMIBC still remain a controversial issue. Recent literature suggested a favorable long-term outcome for timely radical treatment in the case of initially recurrent T1 tumor stage and therapy-refractory^13,23^. The issue whether secondary MIBC predicts a poorer prognosis than primary MIBC is essential. Should the scenario be true, greater emphasis should be given to the debate of timely RC in the management of NMIBC^13^. Therefore, we performed a systematic review and meta-analysis to assess prognostic differences between patients with primary MIBC and patients with secondary MIBC. We also discussed whether appropriate selection criteria existed to risk-stratify the secondary MIBC patients, as well as the potential mechanism behind the difference between secondary MIBC and primary MIBC.

## Results

### Study Characteristics

In total, 2,469 papers based on the search concepts were initially identified. Supplementary Fig. S1 displays the article selection process used in this study (see Supplementary File). In total, 14 comparative, nonrandomized, observational studies^3,4,6–8,10–15,17,18,20^ were included in the final systematic review and meta-analysis (Tables 1,2). The eligible studies in this present study were published between 2002 and 2016. Among the studies, 8 had originated from Europe, 3 from North America, and 3 from Asia. The total number of patients of 13 studies was 4,075 (range: 55–1,150; ref.^4^ was not included)^3,6–8,10–15,17,18,20^. Overall, the ratio between patients with primary MIBC and those with secondary MIBC was 2.3.

**Table 1:** Characteristics of studies included in this meta-analysis.

No.	Author	Country	Period	Year	Study design	primary	secondary	No. of patient	Gender(m/f)
1	Yiou	France	1987–1997	2002	Retrospective	43	12	55	NA
2	Schrier	Netherlands	1986–2000	2004	Retrospective	89	74	163	125/38
3	Lee YH	Korea	1986–2004	2007	Retrospective	173	50	223	200/23
4	Turkolmez	Turkey	1990–2005	2007	Retrospective	109	45	154	134/20
5	Lee	USA	1990–2003	2007	Retrospective	169	70	239	182/57
6	de Vries	Netherlands	1987–2005	2010	Retrospective	134	54	188	144/44
7	Rodriguez	Spain	1978–2002	2011	Retrospective	72	69	141	116/25
8	Kotb	Canada	NA	2012	Retrospective	785	365	1150	914/235
9	Masson-Lecomte	France	2001–2011	2013	Retrospective	155	24	179	166/25
10	Hidas	Israel	1998–2008	2013	Retrospective	104	40	144	112/32
11	Aziz	Germany	2004–2010	2013	Prospective	125	25	150	121/29
12	May	Germany	1992–2007	2014	Prospective	399	122	521	388/133
13	Breau	Canada	1980–1998	2014	Retrospective	481	190	671	512/159
14	Moschini	Italy	2000–2012	2016	Retrospective	475	293	768	641/127

**Table 2:** Characteristics of studies included in this meta-analysis (cont).

No.	Author	Mean age(yr)	Mean FU(months)	Surveillance time of primary MIBC(months)	Quality scale (stars)^*^
1	Yiou	Primary:62 Secondary:66	Primary:49 Secondary:55.3	57	4
2	Schrier	Primary:63.3 Secondary:68.5	NA	NA	5
3	Lee YH	62	45	15	5
4	Turkolmez	Primary:59.8 Secondary:60.3	Primary:77.8 Secondary:90.3	41.7	6
5	Lee	Primary:65 Secondary:69	Primary:40(median) Secondary:33(median)	48	6
6	de Vries	61	3.4(yr)	NA	5
7	Rodriguez	63(median)	42.5	NA	3
8	Kotb	Primary:66.7 Secondary:67.2	NA	NA	4
9	Masson-Lecomte	Primary:66.8 Secondary:68	NA	36	—
10	Hidas	Primary:72.7 Secondary:69.3	Primary:40.1 Secondary:52.6	44	3
11	Aziz	Primary:69 Secondary:71	46(median)	539(d)	6
12	May	Primary:64.1 Secondary:68.7	65	661(d)	7
13	Breau	Primary:67.9 Secondary:67.6	NA	1.8(yr)	5
14	Moschini	Primary:68 Secondary:67	109	NA	6

NA: not available; MIBC: muscle-invasive bladder cancer; FU: follow-up.

^*^Number of stars.

Note that refs^4,12^ were potentially based on the same patient population. Therefore, according to our eligibility criteria, we included the two studies to perform pooled analyses separately in different models (model I and model II). Model I included 13 studies (ref.^4^ was included as the most “recent” study)^3,4,6–8,10,11,13–15,17,18,20^. Model II also included 13 studies (ref.^12^ was included as the most “complete” study)^3,6–8,10–15,17,18,20^.

### Quality Assessment

For quality assessment, the median quality star was 5 of the 13 eligible studies (mean: 5, range: 3–7). Only 1 study^14^ obtained stars of 7 or more, indicating that it was of high quality (Table 2).

### Cancer-specific Survival

#### Meta-analysis

**Figure 1:**
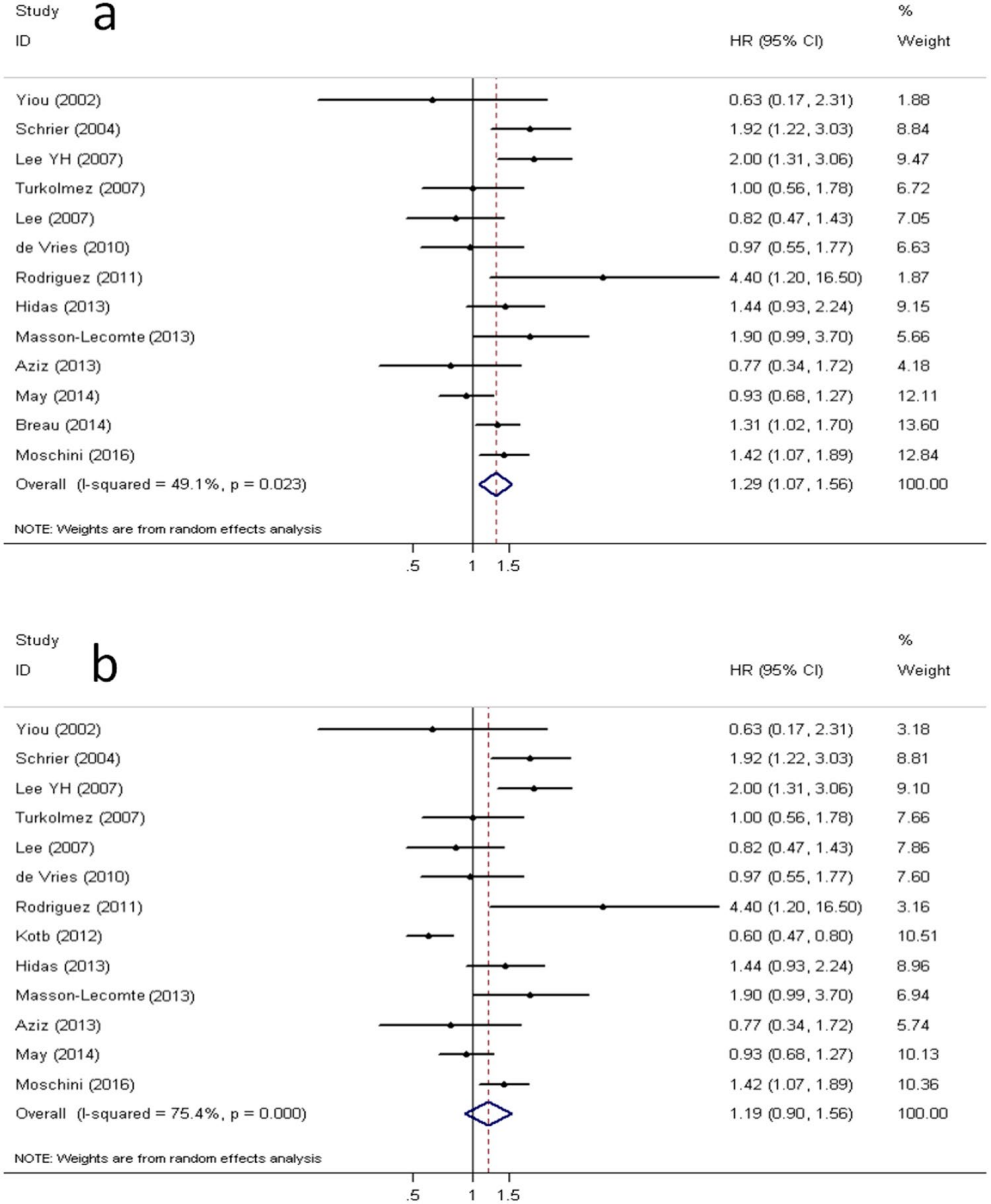
Forest plots. Forest plots show the pooled hazard ratio (HR) from random-effects model for cancer-specific survival according to model I (**a**) and model II (**b**).

Model I: Figure 1a depicts a forest plot of the individual hazard ratio (HR) and results from this meta-analysis. From the pooled analysis, we found that patients with secondary MIBC were significantly correlated with worse cancer-specific survival (CSS) (pooled HR: 1.29, 95% CI: 1.07–1.56, *P* = 0.008). A significant heterogeneity could not be excluded according to Cochrane Q test (Chi^2^ = 23.64, *P* = 0.02) and test of inconsistency (*I*^2^ = 49.2%).

Model II: As is shown in Fig. 1b, 13 studies produced a combined estimate of HR of 1.19, again indicating a worse prognosis for secondary MIBC compared to primary MIBC. However, the difference was insignificant (95% CI: 0.90–1.56, *P* = 0.22).

#### Subgroup Analysis

We further conducted subgroup analyses to evaluate if there were differences in results by publication year, region, population size, methodological quality scales, and the HR estimation in model I and model II. The pooled HR for almost all subgroup analyses again supported the notion that secondary MIBC patients had a poorer survival, when compared with primary MIBC patients (pooled HRs > 1). However, most of results did not yield any significant difference (HR: 95% CIs overlap 1, *P* > 0.05, see Supplementary Table S1, S2).

#### Sensitivity Analyses

We conducted sensitivity analyses to determine the influence of individual study upon the overall effect through omitting a single study each time.

Model I: The overall HRs and 95% CIs were not significantly changed when any one of the 13 studies was excluded, which indicated that there was no single sensitive study and confirmed the robustness of the pooled results in this meta-analysis (see Supplementary Fig. S2).

Model II: As is shown in Supplementary Fig. S3, all the pooled HRs were still greater than 1(point estimation). However, the overall HRs and 95% CIs were significantly changed when one study was omitted (pooled HR = 1.29, 95% CI: 1.03–1.61)^12^. In that case, the result of overall analysis was in keeping with those in model I. Through a further analysis, we found the study was potentially an outlier (See publication bias part).

#### Publication Bias

Publication bias was evaluated using funnel plots and Egger’s test.

**Figure 2:**
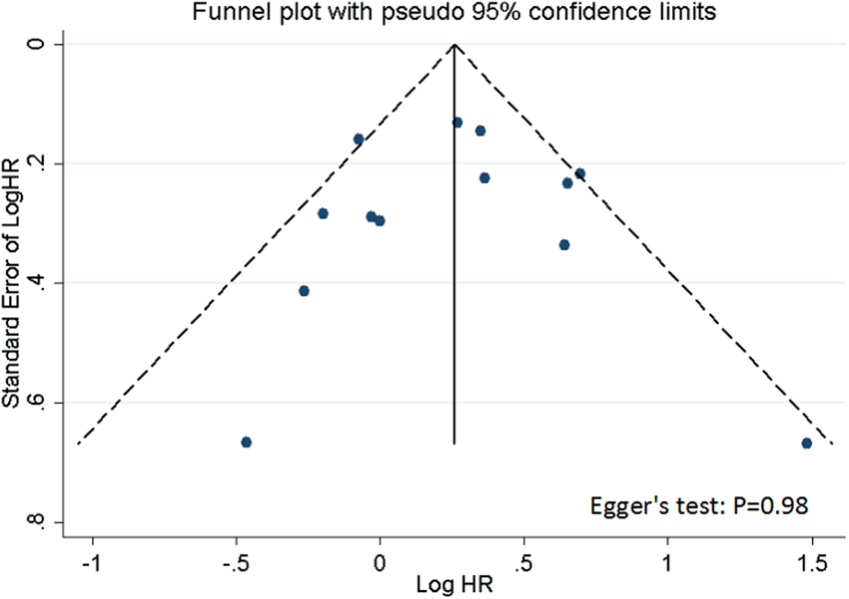
Funnel plots. Funnel plots with pseudo 95% confidence limits for publication bias test according to model I.

Model I: No evidence of obvious asymmetry of the funnel plot in the overall analysis was found, and this observation was further confirmed by Egger’s test (t = -0.02, *P* = 0.98, Fig. 2).

**Figure 3:**
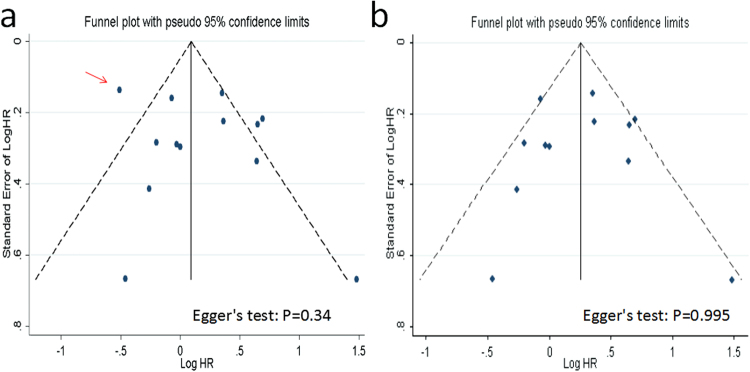
Funnel plots. Funnel plots with pseudo 95% confidence limits for publication bias test according to model II. (**a**) ref.^12^ (red arrow) is included; (**b**) ref.^12^ is excluded.

Model II: We observed a certain degree of asymmetry of the funnel plot, which indicated a slight potential publication bias (Fig. 3a). When the sensitivity analyses above were taken into consideration, one study (ref.^12^) was likely to lead to asymmetry. After the study was omitted and then a reanalysis was made, no obvious asymmetry of the funnel plot was found (Egger’s test: t = 0.01, *P* = 0.995, Fig. 3b). In addition, heterogeneity was also slightly improved (Chi^2^ = 23.63, *P* = 0.01; *I*^2^ = 53.4%).

## Discussion

This is a systematic review and meta-analysis to assess prognostic differences between patients with primary MIBC and those with secondary MIBC. The present study showed that secondary MIBC conveyed an inferior outcome compared to primary MIBC.

Historically, RC was primarily indicated for MIBC patients^10^. This fostered a clinical practice pattern of prolonged use of conservative treatment with closer surveillance for NMIBC before undergoing RC^10^. Theoretically, when used optimally, surveillance can not only allow patients to keep a functional bladder, but also provide a survival advantage, intuitively because the tumor is detected early^10^. Despite this assumption, the results of this present study suggested that detection of MIBC under surveillance provided no survival benefit to primary MIBC. What’s more, we found that the prognosis of secondary MIBC indicated an adverse survival, with a combined HR of 1.29 (95% CI: 1.07–1.56) according to model I and 1.19 (95% CI: 0.90–1.56) according to model II, respectively.

Note that the findings regarding model I (including ref.^4^ and model II (including ref.^12^) are different and model II was not significant (*P* = 0.22). Through a further analysis, we revealed that the study (ref.^12^) might be an outlier in model II. After the study was omitted and then a reanalysis was followed, in analogy to model I the pooled HR (1.29, 95% CI: 1.03–1.61) again confirmed a significantly worse prognosis for secondary MIBC, showing a statistically significant difference (Fig. S3). One possible reason may account for the discrepancy in the findings. Although both of the two studies included Canadians, ref.^12^ was analyzed based on data collected from eight centers across Canada (including University of Ottawa) and ref.^4^ was only based on one center (University of Ottawa). In the former study^12^, most cases lacked pathologic and clinical specifics regarding their prior NMIBC histories, thus resulting in potential selection bias.

Since secondary MIBC obtained an inferior outcome to primary MIBC as showed in this study, could one naturally conclude that greater emphasis should be placed upon the debate concerning early RC in the management of “all” NMIBC patients? Probably not. Except the organ-preservation purposes, another fact that should be taken into consideration is that the NMIBC is not a homogeneous entity. The risk of tumor progression is associated with several factors: high grade, pT1 stage, multifocal tumors^24^, tumor of >3 cm^24^, presence of *in situ* carcinoma^24^, incomplete remission^25^, and gene P53 expression. Difficulties arise in the identification of high-risk patients, who may likely fail conservative therapy, and whose NMIBC disease will progress to invasive, thus requiring RC. Because the prognosis will worsen after MIBC, debate of early RC should be stressed in high-risk NMIBC prior to MIBC.

Tumor stage progression of NMIBC to secondary MIBC is an undisputable indication for RC^26^. However, it seems that not all the patients with secondary MIBC have the same prognosis^11,13,14^. May *et al*.^14^ and Aziz *et al*.^13^ stratified the secondary MIBC patients into different risk groups according to the European Organization for Research and Treatment of Cancer (EORTC) risk score, which is popularly used in predicting disease recurrence and progression in patients with NMIBC^24^. Both studies indicated that high EORTC risk score reflected a worse outcome after RC, which underlined the poor outcome of the high-risk NMIBC group, thus providing further weight to arguments for early cystectomy^11^. de Vries *et al*.^11^ who used European Association of Urology (EAU) risk-categories similar to EORTC risk score also affirmed this conclusion. Similarly, several studies demonstrated that delayed RC for patients with high-risk NMIBC reduced CSS^23^. In some high-risk NMIBC cases, muscular invasion is inevitable, in part for quality of life reasons. What’s more, it is intuitively reasonable to consider that the prognosis of secondary MIBC may be poorer than the results obtained in this meta-analysis, partly because NMIBC patients with high risk disease have undergone RC before muscular invasion and the “residual” secondary MIBC are relatively “benign”. Taken together, there is a great need for further investigations stressing on assessing the multimodal therapy in both high-risk NMIBC and high-risk secondary MIBC patients^14^.

Numerous studies have demonstrated that delayed RC from the diagnosis of MIBC resulted in a worse prognosis^27–30^. Another question that should be stressed is whether patients with secondary MIBC are more likely to have the misfortune of delayed RC^13,14^. May *et al*.^14^ reported that patients with secondary MIBC were less likely to have RC in time: 28.7% of patients with secondary MIBC and 12.5% of patients with primary MIBC underwent delayed RC for >3 months from the diagnosis of MIBC (*P* < 0.01). Aziz *et al*.^13^ claimed that 12.0% of patients with secondary MIBC underwent delayed RC, while for patients with primary MIBC the proportion was only 5.6% (*P* = 0.37). In clinical practice, clinicians should be wary of the pejorative impact of delayed RC on survival and further alert that patients with secondary MIBC can possibly more tend to have this misfortune. Conceivably, in these circumstances, neoadjuvant systemic chemotherapy should be taken into consideration, for it has been shown to improve survival for patients with MIBC^4^. However, in a recent study, Pietzak *et al*.^31^ reported that secondary MIBC was associated with lower response rates to neoadjuvant systemic chemotherapy compared to primary MIBC. Though no solid conclusion could be drawn because of the limited studies, this again suggested a difference between secondary MIBC and primary MIBC.

In addition, our understanding of MIBC should not be limited to primary and secondary patterns. The underlying genetic and molecular drivers of tumor induction, promotion, and progression also need to be investigated. Two hypotheses may help us understand the aggressive pattern of secondary tumors. First, intravesical therapies and cytotoxic cancer therapies given to patients with NMIBC may counter-productively select for the propagation of resistant clones and/or cancer stem cells, and such special tumor cells may play a role in the development of progressive tumor^7,17^. This hypothesis is indirectly supported by the basic studies, which indicate that cancer stem cells contribute to the progression of bladder cancer^17,32,33^. Second, transurethral resections of the bladder tumor (TURBT) causes intravesical tumor cell spreading not only intravesically but also hematogenously. There is no doubt that secondary MIBC patients undergo more TURBTs than primary MIBC patients. El-Abbady *et al*.^22^ compared 16 patients with secondary MIBC to 20 patients diagnosed with primary MIBC through a thorough histopathological study. They found that when compared to patients with primary MIBC, patients who underwent TURBTs had a significantly more local spread of malignant cells into the bladder muscle through the denuded urothelium as a result of high intravesical pressure during TURBT. Interestingly and potentially similarly, in a more recent retrospective series, Wiesner *et al*.^34^ observed that the number of TURBTs increased the prevalence of lymph node metastases from 8% in patients with only one TURBT to 24% in those with two to four TURBTs. In the most recent finding, Blaschke *et al*.^35^ measured circulating tumor cells before and after TURBT in seven cases of confirmed urothelial carcinoma. In two patients with MIBC, circulating tumor cells were detectable after but not before TURBT. The authours concluded that the TURBT technique might involve risks in hematogenous tumor cell spreading. Nevertheless, the underlying reason still remains a mystery.

We believe our study offers new information in answering the question of prognostic differences between patients with primary MIBC and patients with secondary MIBC. However, it does bear potential limitations that need to be acknowledged. The first limitation was a small number of studies, which hampered firm conclusions. Especially in subgroup analyses, though the pooled HRs again supported the notion that secondary MIBC patients had a poorer survival (pooled HRs > 1), most of results did not yield any statistically significant difference (95% CIs overlap 1). Note that subgroup analysis in model 1 indicates that studies that used a multivariate approach had a significant pooled HR. The non-statistically significant results may partly result from the limited number of included studies. There is a great need for more studies evaluating the prognosis of secondary MIBC on survival.

Another weakness of our study was the heterogeneity. Although heterogeneity was taken into consideration by using the random effect model, studies have differed with regard to the baseline characteristics of patients, surgical techniques, and follow-up schedules; the aforementioned factors may lead to discrepancy in the findings. Specifically, considering that study 12^12^, which was a multicenter study with heterogeneity in disease severity and subsequent management, may be an outlier in model II, this weakness should be taken into consideration.

Furthermore, we used the NOS to assess the included studies in this investigation. Because no standard validated criteria for important end points have been established, we “empirically” considered a study awarded seven or more stars to be of high quality. However, according to this criterion, most of the studies have low to moderate scores, which may thus account for biased results and compromise the strength of their conclusion. From another perspective, it should be realized that although the NOS is popularly used in evidence-based reviews and meta-analyses, the use of this score still remains controversial and may even produce highly arbitrary results^36,37^. In fact, there is no clear consensus on quality assessment of observational studies. In order to guarantee the minimum possible effect of studies of poor quality, we established strict inclusion and exclusion criteria before reviewing the studies and extracting the data.

Thus, the conclusion drawn in this meta-analysis should be interpreted with caution.

## Methods

### Search Strategy

In general, the meta-analysis study is exempt from ethics approval. We conducted and reported this study following the PRISMA statement^38^. Comprehensive electronic searches of the Medline (on ovid), Embase (on ovid), the Cochrane Library, and Scopus databases were performed (A detailed search strategy is presented in Supplementary File). The last search was conducted in January 2017. No language or other restrictions were imposed on the searches. References from retrieved articles relating to our study topic were reviewed and cross referenced to ensure completeness of our literature search.

### Inclusion and Exclusion Criteria

A study was selected for analysis if they met all of the following eligibility criteria^39^: (1) the study included proved diagnosis of bladder cancer; (2) the study assessed prognostic differences between patients with primary and those with secondary MIBC; (3) the number of patients in each group should be not less than 10; (4) the study reported a HR with 95% confidence interval (CI) directly or reported the data that allows for calculation; Exclusion criteria were: (1) review papers, letters to the editor, replies, book chapters, commentaries, conference abstracts, or case report; (2) basic studies, such as studies on cell lines and animal models; (3) duplicate publications. All studies were carefully checked to avoid duplicate data. When more than one publication reported outcomes for the same patient population, the most complete and the most recent study were analyzed in separate models. Two investigators independently selected studies and discussed with each other when inconsistencies were found (P.G. and L.W.).

### Data Collection

For each eligible study, two authors(P.G. and L.W.) independently extracted the following data^39^: (1) publication data encompassing the author’s last name, year of publication, country (patient populations), study design, period of recruitment, and population size; (2) clinicopathological data such as age, gender, follow-up period, surveillance time of secondary MIBC, pathological type, pathological grade, pathological T/N/M stage; (3) statistical data including HRs and their CIs. Surveillance time of primary MIBC was defined as the time from the initial TURBT to cystectomy or last TURBT, and also defined as the time from the diagnosis of NMIBC to MIBC. If the HR was not presented directly, available survival data from original articles were used to estimate the HR by using the methods described previously^40,41^. The Engauge Digitizer version 4.1 was used to read the Kaplan–Meier survival curves (free software, available: http://sourceforge.net). In studies for which both univariate and multivariate analyses were available, the multivariate results were used to calculate HRs and CIs. Inconsistencies were resolved by discussion.

### Methodological Assessment

The Newcastle-Ottawa Scale (NOS)^42^ was introduced to evaluate methodological quality of the included studies. Using a star system, a study is assessed based on the selection of study subjects (maximum: four stars), comparability of study groups (maximum: two stars) and outcome assessment (maximum: three stars). In this study, we considered a study awarded seven or more stars to be of high quality, because no standard validated criteria for important end points have been established.

### Statistical Analysis

CSS was the outcome measure. The log-HRs and 95% CIs from each study were obtained and subsequently were used to conduct this meta-analysis (HR relates to CSS). According to a priori assumptions about the likelihood for heterogeneity across studies, all pooled outcome measures were determined using the random-effects models (the DerSimonian and Laird)^43^. By convention, an observed HR > 1 indicated an adverse survival for the secondary MIBC group, relative to the primary MIBC group (reference). If *P* < 0.05 or the 95% CI did not overlap 1, the pejorative impact of secondary MIBC on outcome was considered to be statistically significant. The chi-square–based Cochrane Q statistics test was used to assess heterogeneity across the studies included in this meta-analysis. A *P* < 0.10 for the Q test indicated heterogeneity cross the studies. *I*^2^ was also used to test the magnitude of the between-study heterogeneity^44,45^. To assess the robustness of pooled HRs and to analyze the source of heterogeneity, the subgroup analysis was performed through the stratification by publication year, region, population size, methodological quality scales, and HR estimation^39^. Furthermore, sensitivity analyses were performed to assess the stability of results by deleting a single study each time. The potential publication bias was evaluated visually in a funnel plot, and the degree of asymmetry was evaluated by Egger’s test^46^.

Statistical analyses were all performed using Stata statistical software (version 12.0, Stata Corp, College Station, TX). All *P*-values were based on two-sided tests and a *P*-value of less than 0.050 was considered statistically significant.

## Electronic supplementary material

Supplementary File
