# Supplementary material for: Oncological Outcome of Primary and Secondary Muscle-Invasive Bladder Cancer: A Systematic Review and Meta-analysis
Source: Sci Rep. 2018 May 15;8:7543. doi: 10.1038/s41598-018-26002-6 (PMC5954122; doi:10.1038/s41598-018-26002-6)
Supplement: Supplementary file 1 — Supplementary File [file 41598_2018_26002_MOESM1_ESM.pdf]

Oncological Outcome of Primary and Secondary Muscle-Invasive Bladder Cancer: A Systematic Review and Meta-analysis

Peng Ge[1,+,*], Li Wang[2,+], Meng Lu[1], Lijun Mao[1], Wang Li[1], Rumin Wen[1], Jian Lin[3,4], Junqi Wang[1,*] and Jiacun Chen[1,*]

[1]Department of Urology, the affiliated hospital of Xuzhou Medical University, Xuzhou, Jiangsu, China.

[2]Xuzhou Medical University, Xuzhou, Jiangsu, China.

[3]Department of Urology, Peking University First Hospital and Institute of Urology, Peking University, Beijing, China.

[4]National Research Center for Genitourinary Oncology, Beijing, China.

[+]These authors contributed equally to this work.

*Correspondence and requests for materials should be addressed to J.W. (email: wjq68@sina.cn) or J.C. (email: cjc7812@163.com) or P.G. (gepengyzu@126.com)

Contents

Figure S1 ... 3

More information of full-text articles excluded ... 4

Table S1 ... 11

Table S2 ... 12

Figure S2 ... 13

Figure S3 ... 14

Search strategy ... 15

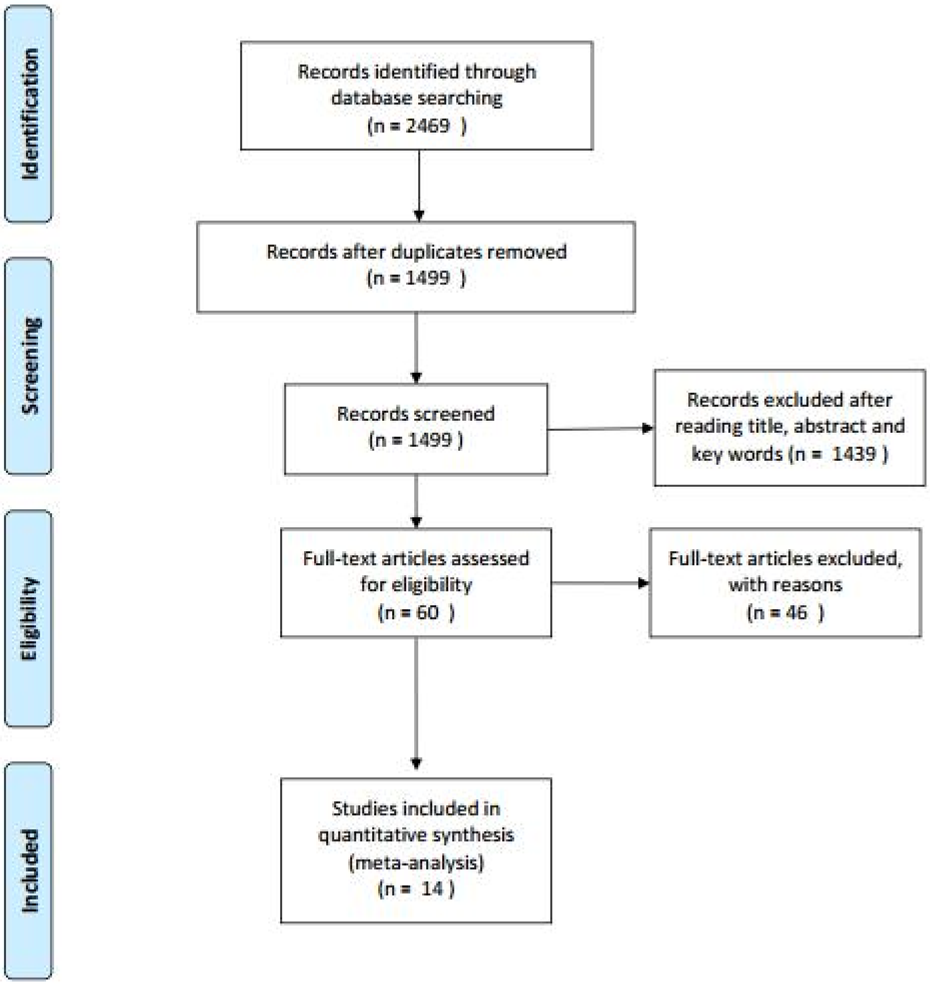

Figure S1 Flow chart of the literature search used in this meta-analysis. Of those studies, two studies (reference 6 and 15 in main text) were from a same medical center, in which data was not duplicate, and these studies were all include for pooled analysis

More information of full-text articles excluded

Abbreviation: MIBC, muscle-invasive bladder cancer; CSS, Cancer-specific survival; OS, overall survival; RFS, recurrence-free survival; HR, hazard ratio; CI, confidence interval.

No.	Author	Year	Main results	Reason for not including in pooled analysis
1	Brant[1]	2017	Among non-neoadjuvant chemotherapy patients, secondary MIBC was significantly less prevalent in patients who achieved pathologic response compared with those who did not respond (3.6% vs. 25.2%, P＜0.01).	Inadequate information. CSS was not available.
2	Zakaria[2]	2016	Presumed secondary MIBC had a slightly better prognosis, regarding OS.	Histopathologic or staging clinical information was lacking and primary and secondary MIBC were indirectly presumed. CSS was not available.
3	Pietzak[3]	2016	-	Abstract
4	Brant[4]	2016	-	Abstract
5	Moschini[5]	2016	-	Abstract
6	Mallen[6]	2015	-	Inadequate information. CSS was not available.
7	Pietzak[7]	2015	-	Abstract
8	Pietzak[8]	2015	Secondary MIBC was associated with upstaging and had a worse OS.	Inadequate information. CSS was not available.
9	Parra-Lopez[9]	2014	-	Abstract
10	Pellucchi[10]	2014	-	Abstract
11	Alexandra[11]	2013	-	Abstract

12	Koga[12]	2013	-	Irrelevant topic
13	Husillos-Alonso[13]	2013	Secondary MIBC had a similar tumor progression rate compared to primary MIBC (24.1% v.s. 23.5%).	Inadequate information.
14	Ma[14]	2013	MIBC with prior history of urothelial carcinoma was associated with both CSS (HR 3.235, CI 1.211-8.644, P=0.019) and RFS (HR 2.713, CI 1.231-5.978, P=0.013) and weakly associated with OS (P=0.064).	MIBC with prior history of urothelial carcinoma was not exactly equal to secondary MIBC. **If this study was included in pooled analysis, the conclusion drawn in the study would be further confirmed.**
15	Ciudin[15]	2012	-	Abstract
16	Ciudin[16]	2012	-	Abstract
17	Ciudin[17]	2012	-	Abstract
18	Ciudin[18]	2011	-	Abstract
19	Ploeg[19]	2011	-	Inadequate information.
20	Villavicencio[20]	2010	-	Less than 10 patients in secondary MIBC group and/or primary MIBC.
21	Brookman-Amissah[21]	2009	-	Abstract
22	Guzzo[22]	2009	-	Abstract
23	Huguet[23]	2008	-	Irrelevant topic

24	Ferreira[24]	2007	Secondary MIBC had similar prognosis compared to primary MIBC (5 years survival: 57.9% v.s. 52.2%).	Data is inconsistency.<br>According to the study, patients between 1993 and 2005 were included and manuscript was accepted for publication on October 11, 2006. Theoretically, the maximum follow-up time should be less than 166 months. However, figure 1 suggests that the maximum follow-up time is more than 180 months.<br>**If this study was included in pooled analysis, no significant changes would be observed.**
25	May[25]	2007	-	Duplicate publication
26	McLaughlin[26]	2007	-	Irrelevant topic
27	Louie-Johnsun[27]	2007	-	Irrelevant topic
28	Kassouf[28]	2006	-	Less than 10 patients in secondary MIBC group and/or primary MIBC.
29	Jeon[29]	2005	-	Less than 10 patients in secondary MIBC group and/or primary MIBC.
30	May[30]	2004	-	Duplicate publication
31	Hornak[31]	2004	Secondary MIBC had worse prognosis compared to primary MIBC (3 years survival: 15.4% v.s. 23%).	Full article is not available.<br>The study is in Slovak. We reqested for full-text through ResearchGate, but no reply was received.
32	Knap[32]	2003	Patients with primary invasive in lamina propria (Tl) progressing to MIBC and with primary non-invasive (Ta. CIS) progressing to MIBC had a significantly poorer outcome compared to primary MIBC.	Inadequate information.<br>The prognosis of the overall secondary MIBC was not clear.

33	Horňak[33]	2003	-	Duplicate publication
34	Larsson[34]	2003	-	Inadequate information.
35	Yeo[35]	2002	Primary MIBC had higher tumor grade and lower five-year survival rate compared to secondary MIBC ($P < 0.05$).	Data is inconsistency. According to the patients&methods part, the follow-up time was ranging from 36 to 98 months. However, figure 1 suggests that the minimum follow-up is less than 24 months. **If this study was included in pooled analysis, no significant changes would be observed.**
36	Vaidya[36]	2001	-	Inadequate information.
37	Huguet-Perez[37]	2001	-	Less than 10 patients in secondary MIBC group and/or primary MIBC.
38	Bassi[38]	1999	-	Inadequate information.
39	Irani[39]	1997	-	Inadequate information.
40	Fossa[40]	1992	-	Inadequate information.
41	Stockle[41]	1988	-	Inadequate information.
42	Malmstrom[42]	1987	-	Inadequate information.
43	Droller[43]	1983	-	Less than 10 patients in secondary MIBC group and/or primary MIBC.
44	Hopkins[44]	1983	-	Less than 10 patients in secondary MIBC group and/or primary MIBC.
45	Kaye[45]	1982	-	Inadequate information.
46	Boileau[46]	1980	-	Inadequate information.

References:

1. Brant, A., *et al.*. Pathologic response in patients receiving neoadjuvant chemotherapy for muscle-invasive bladder cancer: Is therapeutic effect owing to chemotherapy or TURBT? *Urol Oncol* **35**, 17-34 (2017).
2. Zakaria, A.S., Santos, F., Kassouf, W., Tanguay, S. & Aprikian, A. Survival after Radical Cystectomy for Bladder Cancer in Relation to Prior Non-Muscle Invasive Disease in Quebec. *Urol Int* (2016).
3. Pietzak, E., *et al.*. Progression from non-muscle invasive bladder cancer (NMIBC) to muscle invasion is associated with lower response rates to neoadjuvant chemotherapy. *J Urology* **195S**, E408-E409 (2016).
4. Brant, A., *et al.*. Effect of chemotherapy and/or TURBT on pathologic response in patients receiving neoadjuvant chemotherapy for muscle-invasive bladder cancer. *J Clin Oncol* **34**, no pagination (2016).
5. Moschini, M., *et al.*. Preoperative favourable characteristics in bladder cancer patients cannot substitute the necessity of extended lymphadenectomy during radical cystectomy: A sensitivity curve and a survival analysis. *European Urology, Supplements* **15**, e627 (2016).
6. Mallen, E., Gil, P. & Gil, M.J. Risk groups in bladder cancer patients treated with radical cystectomy. *Int Braz J Urol* **41**, 30-39 (2015).
7. Pietzak, E.J., Malkowicz, S.B. & Guzzo, T.J. Does "low risk" muscle invasive bladder cancer actually exist? *J Urol* **33**(2015).
8. Pietzak, E.J., Sterling, M.E., Smith, Z.L., Malkowicz, S.B. & Guzzo, T.J. Outcomes of radical cystectomy in potential candidates for bladder preservation therapy. *Urology* **85**, 869-875 (2015).
9. Parra-Lopez, L., *et al.*. Prognostic factors and survival differences between primary and progressive muscle-invasive bladder cancer. *European Urology, Supplements* (2014).
10. Pellucchi, F., *et al.*. Progression of T1 high risk into muscle-invasive bladder cancer is an independent prognostic factor of mortality after radical cystectomy. *J Urol* **191**, e685-e686 (2014).
11. Alexandra, M.L., *et al.*. Oncological outcome of radical cystectomy for BCG failure compared to primary invasive disease. *J Urol* **1)**, e666 (2013).
12. Koga, F., *et al.*. Risk factors for intravesical tumor recurrence in muscle-invasive bladder cancer patients treated with selective bladder-sparing approaches. *European Urology, Supplements* **12**, e76-e77 (2013).
13. Husillos-Alonso, A., *et al.*. Prognostic factors in patients with stage pT0/pT1/pTa in the radical cystectomy specimen. *Arch Esp Urol* **66**, 787-795 (2013).
14. Ma, B., *et al.*. Lymphovascular invasion, ureteral reimplantation and prior history of urothelial carcinoma are associated with poor prognosis after partial cystectomy for muscle-invasive bladder cancer with negative pelvic lymph nodes. *Eur J Surg Oncol* **39**, 1150-1156 (2013).

15. Ciudin, A., Huguet, J., Peri, L., Ribal, M.J. & Alcaraz, A. T4 bladder tumour affecting the prostate. Prognostic differences depending on the bladder tumour evolution: Primary versus progressive tumours. *European Urology, Supplements* **11 (5)**, 199-200 (2012).
16. Ciudin, A., Huguet, J., Musquera, M., Ribal, M.J. & Alcaraz, A. Differences in patterns of prostatic stromal involvement between primary and progressive muscle invasive bladder tumours. *European Urology, Supplements* **11 (1)**, e719 (2012).
17. Ciudin, A.*, et al.*. Prognosis of primary and progressive muscle-invasive urothelial bladder carcinoma - Is there a difference? *European Urology, Supplements* **11 (5)**, 199 (2012).
18. Ciudin, A.*, et al.*. Prognosis of primary and progressive muscle-invasive urothelial bladder carcinoma - Is there a difference? *European Urology, Supplements* **10 (2)**, 241 (2011).
19. Ploeg, M.*, et al.*. Prognostic factors for survival in patients with recurrence of muscle invasive bladder cancer after treatment with curative intent. *Clin Genitourin Cancer* **9**, 14-21 (2011).
20. Villavicencio, H.*, et al.*. Bladder preservation strategy based on combined therapy in patients with muscle-invasive bladder cancer: management and results at long-term follow-up. *Urol Int* **85**, 281-286 (2010).
21. Brookman-Amissah, S. & May, M. Radical cystectomy in the treatment of bladder cancer: Always in due time? *European Urology, Supplements* (2009).
22. Guzzo, T.J.*, et al.*. Pathologic upstaging at the time of radical cystectomy is associated with worse recurrence free survival in patients with BCG-refractory bladder cancer. *J Urol* **1)**, 641 (2009).
23. Huguet, J.*, et al.*. Diagnosis, risk factors, and outcome of urethral recurrences following radical cystectomy for bladder cancer in 729 male patients. *Eur Urol* **53**, 785-792 (2008).
24. Ferreira, U.*, et al.*. Primary invasive versus progressive invasive transitional cell bladder cancer: multicentric study of overall survival rate. *Urol Int* **79**, 200-203 (2007).
25. May, M.*, et al.*. Radical cystectomy in the treatment of bladder cancer always in due time? *Urologe A* **46**, 913-919 (2007).
26. McLaughlin, S.*, et al.*. Comparison of the clinical and pathologic staging in patients undergoing radical cystectomy for bladder cancer. *Int Braz J Urol* **33**, 25-31, 31-32 (2007).
27. Louie-Johnsun, M.W., Braslis, K.G., Murphy, D.L., Neerhut, G.J. & Grills, R.J. Radical cystectomy for primary bladder malignancy: a 10 year review. *Anz J Surg* **77**, 265-269 (2007).
28. Kassouf, W.*, et al.*. Partial cystectomy for muscle invasive urothelial carcinoma of the bladder: a contemporary review of the M. D. Anderson Cancer Center experience. *J Urol* **175**, 2058-2062 (2006).
29. Jeon, S.H., Jeon, S.H. & Chang, S.G. Clinical prognostic factors for radical cystectomy in bladder cancer. *Cancer Res Treat* **37**, 48-53 (2005).

30. May, M., Helke, C., Nitzke, T., Vogler, H. & Hoschke, B. Survival rates after radical cystectomy according to tumor stage of bladder carcinoma at first presentation. *Urol Int* **72**, 103-111 (2004).
31. Hornak, M. & Bardos, A. Biologic characteristics of invasive bladder cancer: is there a difference between primary and progressive tumours? *Rozhl Chir* **83**, 648-650 (2004).
32. Knap, M.M., Lundbeck, F. & Overgaard, J. Prognostic factors, pattern of recurrence and survival in a Danish bladder cancer cohort treated with radical cystectomy. *Acta Oncol* **42**, 160-168 (2003).
33. Horňak, M. & Bárdoš, A. Invasive bladder tumours: Analysis of clinical material. *Lekarsky Obzor* **52**, 360-362 (2003).
34. Larsson, P.*, et al.*. A population-based study of 538 patients with newly detected urinary bladder neoplasms followed during 5 years. *Scand J Urol Nephrol* **37**, 195-201 (2003).
35. Yeo, J., Ko, Y., Ko, Y. & Yoon, D. The Prognostic Difference between the Invasive Bladder Cancer at the Initial Diagnosis and the Invasive Bladder Cancer Progressed from Superficial. *Korean J Urol* **2**, 112-116 (2002).
36. Vaidya, A., Soloway, M.S., Hawke, C., Tiguert, R. & Civantos, F. De novo muscle invasive bladder cancer: is there a change in trend? *J Urol* **165**, 47-50, 50 (2001).
37. Huguet-Perez, J.*, et al.*. Upper tract transitional cell carcinoma following cystectomy for bladder cancer. *Eur Urol* **40**, 318-323 (2001).
38. Bassi, P.*, et al.*. Prognostic factors of outcome after radical cystectomy for bladder cancer: a retrospective study of a homogeneous patient cohort. *J Urol* **161**, 1494-1497 (1999).
39. Irani, J.*, et al.*. Clinical stage (t stage) and pathological stage (pt stage) in cystectomy for bladder cancer: which one is the main determinant of survival? *Br J Urol* **80**, 54 (1997).
40. Fossa, S.D., Ous, S., Espetveit, S. & Langmark, F. Patterns of primary care and survival in 336 consecutive unselected Norwegian patients with bladder cancer. *Scand J Urol Nephrol* **26**, 131-138 (1992).
41. Stockle, M., Jacobi, G.H. & Hohenfellner, R. [Importance of early cystectomy for the prognosis of cancer of bladder]. *J Urol (Paris)* **94**, 401-404 (1988).
42. Malmstrom, P.U., Busch, C. & Norlen, B.J. Recurrence, progression and survival in bladder cancer. A retrospective analysis of 232 patients with greater than or equal to 5-year follow-up. *Scand J Urol Nephrol* **21**, 185-195 (1987).
43. Droller, M.J. & Walsh, P.C. Therapeutic efficacy of salvage cystectomy. Do results reflect natural history of bladder cancer? *Urology* **22**, 118-122 (1983).
44. Hopkins, S.C., Ford, K.S. & Soloway, M.S. Invasive bladder cancer: support for screening. *J Urol* **130**, 61-64 (1983).
45. Kaye, K.W. & Lange, P.H. Mode of presentation of invasive bladder cancer: reassessment of the problem. *J Urol* **128**, 31-33 (1982).
46. Boileau, M.A., Johnson, D.E., Chan, R.C. & Gonzales, M.O. Bladder carcinoma: results with preoperative radiation therapy and radical cystectomy. *Urology* **16**, 569-576 (1980).

	No. of studies	Pooled HR (95% CI)	Chi^2 (*P* value)	I^2
Total	13	1.29(1.07-1.56)	23.64(0.02)	49.2%
Publication year				
2002-2011	7	1.33(0.91-1.94)	15.41(0.02)	61.1%
2012-2016	6	1.26(1.03-1.53)	7.66(0.18)	34.7%
Region				
Europe	8	1.29(0.96-1.73)	15.91(0.03)	56.0%
Others	5	1.31(1.01-1.70)	7.55(0.11)	47.0%
No. of patients				
<200	8	1.34(0.99-1.81)	12.02(0.10)	41.8%
⩾200	5	1.25(0.97-1.62)	11.24(0.02)	64.4%
Quality score				
<6	7	1.50(1.17-1.94)	10.52(0.10)	43.0%
⩾6	5	1.05(0.82-1.34)	6.11(0.19)	34.5%
HR estimation				
Univariate	7	1.17(0.95-1.43)	7.93(0.24)	24.4%
Multivariate	6	1.47(1.07-2.03)	12.40(0.03)	59.7%

HR: hazard ratio, CI: confidence interval

Table S1 Subgroup analysis for cancer-specific survival in model I.

	No. of studies	Pooled HR (95% CI)	Chi^2 (*P* value)	I^2
Total	13	1.19(0.90-1.56)	48.77(<0.001)	75.4%
Publication year				
2002-2011	7	1.33(0.91-1.94)	15.41(0.02)	61.1%
2012-2016	6	1.07(0.74-1.56)	26.87(<0.001)	81.4%
Region				
Europe	8	1.29(0.96-1.73)	15.91(0.03)	56.0%
Others	5	1.07(0.65-1.76)	27.04(<0.001)	85.2%
No. of patients				
<200	8	1.34(0.99-1.81)	12.02(0.10)	41.8%
⩾200	5	1.05(0.68-1.61)	30.95(<0.001)	87.1%
Quality scole				
<6	7	1.31(0.79-2.17)	39.87(<0.001)	85.0%
⩾6	5	1.05(0.82-1.34)	6.11(0.19)	34.5%
HR estimation				
Univariate	6	1.11(0.85-1.44)	6.73(0.24)	25.7%
Multivariate	7	1.29(0.83-1.99)	42.03(<0.001)	85.7%

HR: hazard ratio, CI: confidence interval

Table S2 Subgroup analysis for cancer-specific survival in model II.

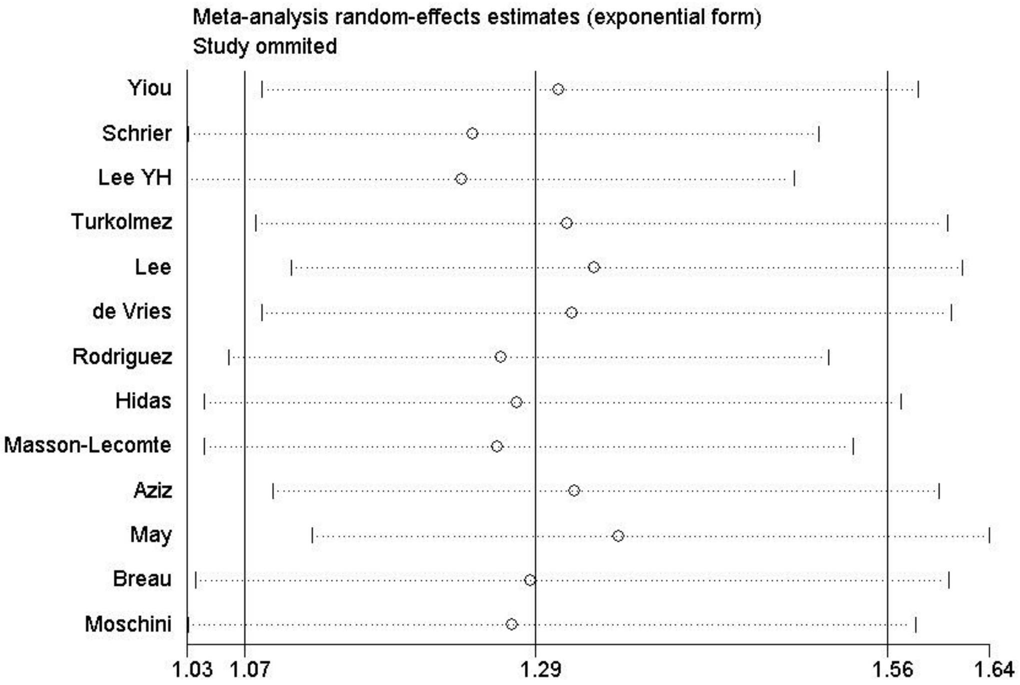

Figure S2 Sensitivity Analyses by omitting a single study each time in model I.

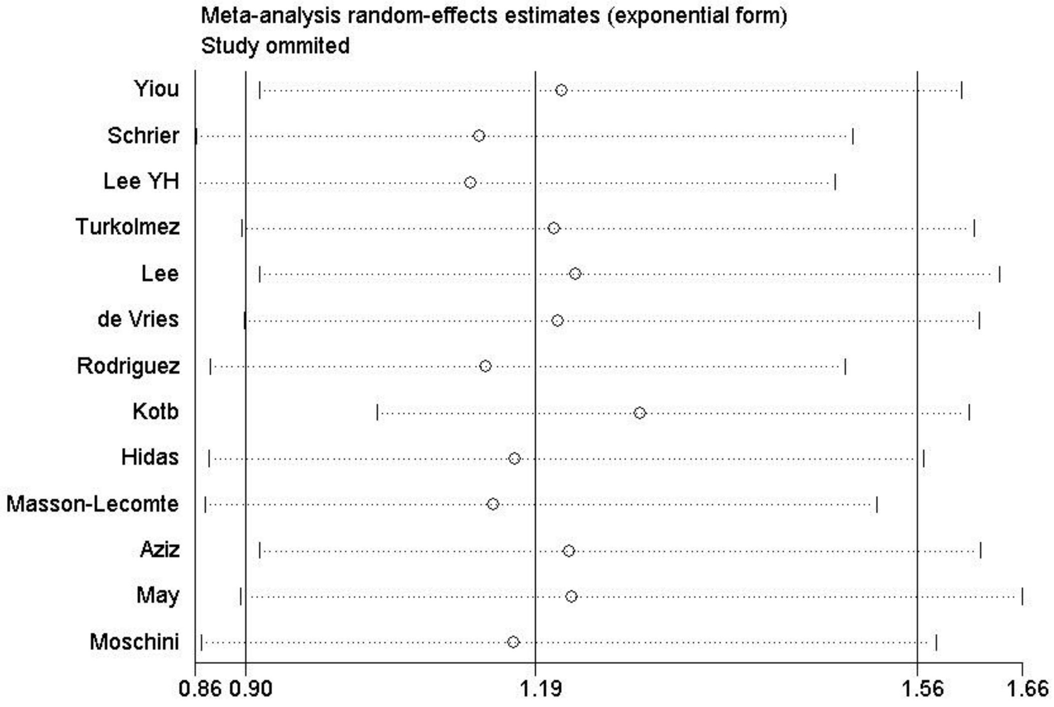

Figure S3 Sensitivity Analyses by omitting a single study each time in model II.

Search strategy

Database: Embase <1974 to 2017 January 03>(on OVID)

1.exp bladder tumor/ (74306)
2. (bladder$ adj3 (cancer$ or carcinoma$ or neoplas$ or tumo?r$)).mp. (81689)
3. exp transitional cell carcinoma/ (22286)
4. (tcc or transitional cell).mp. (28444)
5. or/1-4 (93989)
6.((progress$ or secondary or primar$ or initia$ or previous or prior) adj3 (muscle adj3 invas$)).ti,ab,ot. (747)
7. ((progress$ or secondary or primar$ or initia$ or previous or prior) adj3 (invas$ adj3 (cancer$ or carcinoma$ or neoplas$ or tumo?r$ or malignan$ or disease$ or muscle))).ti,ab,ot. (7778)
8. ((progress$ or secondary or primar$ or initia$) adj3 (MIBC or MIT)).ti,ab,ot. (1012)
9. (muscle adj3 invas$ adj6 (progress$ or secondary or primar$ or initia$ or previous or prior)).ti,ab,ot. (1122)
10. (invas$ adj3 (cancer$ or carcinoma$ or neoplas$ or tumo?r$ or malignan$ or disease$ or muscle) adj6 (progress$ or secondary or primar$ or initia$ or previous or prior)).ti,ab,ot. (11405)
11. ((MIBC or MIT) adj6 (progress$ or secondary or primar$ or initia$)).ti,ab,ot. (1479)
12. secondary muscle-invasive bladder cancer.ti,ab,ot. (6)
13. primary muscle-invasive bladder cancer.ti,ab,ot. (6)
14. or/6-13 (13827)
15. exp cystectomy/ (22339)
16. (rc or cystectom$).mp. (47546)
17. or/15-16 (47546)
18. cancer prognosis/ (66439)
19. exp Survival/ (933156)
20. Treatment Outcome/ (744598)
21. (surival$ or prognos$ or outcome$).ti,ab. (2253231)
22. (os or dfs or css or dss).ti,ab. (129744)
23. or/18-22 (3252047)
24. 5 and 14 and 17 and 23 (522)

Database: MEDLINE(on OVID)

1.exp urinary bladder neoplasms/
2.((bladder$ or urotheli$) adj3 (cancer$ or carcinoma$ or neoplas$ or tumo?r$ or malignan$)).mp.
3.exp carcinoma, transitional cell/
4.bladder neoplasms/
5.(tcc or transitional cell).mp.
6.or/1-5

7.((progress$ or secondary or primar$ or initia$ or previous or prior) adj3 (muscle adj3 invas$)).ti,ab,ot
8.((progress$ or secondary or primar$ or initia$ or previous or prior) adj3 (invas$ adj3 (cancer$ or carcinoma$ or neoplas$ or tumo?r$ or malignan$ or disease$ or muscle))).ti,ab,ot
9.((progress$ or secondary or primar$ or initia$) adj3 (MIBC or MIT)).ti,ab,ot
10.((muscle adj3 invas$) adj6 (progress$ or secondary or primar$ or initia$ or previous or prior)).ti,ab,ot
11.((invas$ adj3 (cancer$ or carcinoma$ or neoplas$ or tumo?r$ or malignan$ or disease$ or muscle)) adj6 (progress$ or secondary or primar$ or initia$ or previous or prior)).ti,ab,ot
12.((MIBC or MIT) adj6 (progress$ or secondary or primar$ or initia$)).ti,ab,ot
13.(secondary muscle-invasive bladder cancer).ti,ab,ot
14.(primary muscle-invasive bladder cancer).ti,ab,ot
15.or/7-14
16.exp cystectomy/
17.(rc or cystectom$).mp.
18.or/16-17
19.exp prognosis
20.exp Survival Analysis
21.Disease-Free Survival/
22.Survival Rate/
23.Treatment Outcome/
24.(surival$ or prognos$ or outcome$).ti,ab.
25.(os or dfs or css or dss).ti,ab.
26.or/19-25
27.6 and 15 and 18 and 26

Database: SCOPUS

((TITLE-ABS-KEY(bladder W/3 tumo?r*) OR TITLE-ABS-KEY(bladder* W/3 cancer*) OR TITLE-ABS-KEY(bladder* W/3 carcinoma*) OR TITLE-ABS-KEY(bladder* W/3 neoplasm*)) OR (TITLE-ABS-KEY(urothelial* W/3 tumo?r*) OR TITLE-ABS-KEY(urothelial* W/3 cancer*) OR TITLE-ABS-KEY(urothelial* W/3 carcinoma*) OR TITLE-ABS-KEY(urothelial* W/3 neoplasm*))) AND (TITLE-ABS-KEY(progres* W/4 invas*) OR TITLE-ABS-KEY(secondary W/4 invas*) OR TITLE-ABS-KEY(previous W/4 invas*) OR TITLE-ABS-KEY(prior W/4 invas*) OR TITLE-ABS-KEY(primary W/4 invas*) OR TITLE-ABS-KEY(initia* W/4 invas*) OR TITLE-ABS-KEY(progres* W/4 muscle) OR TITLE-ABS-KEY(secondary W/4 muscle) OR TITLE-ABS-KEY(previous W/4 muscle) OR TITLE-ABS-KEY(prior W/4 muscle) OR TITLE-ABS-KEY(primary W/4 muscle) OR TITLE-ABS-KEY(initia* W/4 muscle) OR TITLE-ABS-KEY(progres* W/4 MIBC) OR TITLE-ABS-KEY(secondary W/4 MIBC) OR TITLE-ABS-KEY(previous W/4 MIBC) OR TITLE-ABS-KEY(prior W/4 MIBC) OR TITLE-ABS-KEY(primary W/4 MIBC) OR TITLE-ABS-KEY(initia* W/4 MIBC) OR TITLE-ABS-KEY(progres* W/4 MIT) OR TITLE-ABS-KEY(secondary W/4 MIT) OR TITLE-ABS-KEY(previous W/4 MIT) OR TITLE-ABS-KEY(prior W/4 MIT) OR TITLE-ABS-KEY(primary W/4 MIT) OR TITLE-ABS-KEY(initia* W/4 MIT)) AND

(TITLE-ABS-KEY(cystectomy) OR TITLE-ABS-KEY(RC))

Database: Cochrane Library

Cochrane Library

1. MeSH descriptor Urinary Bladder Neoplasms explode all trees
2. (bladder* NEAR/3 (cancer* or carcinoma* or neoplas* or tumo?r*)):ti,ab,kw
3. MeSH descriptor Carcinoma, Transitional Cell explode all trees
4. (tcc or transitional cell):ti,ab,kw
5. #1 OR #2 OR #3 OR #4
6. (progress* or secondary or previous or prior):ti,ab,kw
7. (primar* or initia*):ti,ab,kw
8. (muscle NEAR/4 invas*):ti,ab,kw
9. (invas* NEAR/4 (disease* or transitional cell or muscle or tumo?r*)):ti,ab,kw
10. #8 OR #9
11. #6 AND #7 AND #10
12. (cystectomy or rc):ti,ab,kw
13. MeSH descriptor Cystectomy explode all trees
14. #12 OR #13
15. #5 AND #11 AND #14
